# Thyroid hormones in ovarian follicular fluid: Association with oocyte retrieval in women undergoing assisted fertilization procedures

**DOI:** 10.5935/1518-0557.20200004

**Published:** 2020

**Authors:** Mónica Rosales, Myriam Nuñez, Andrea Abdala, Viviana Mesch, Gabriela Mendeluk

**Affiliations:** 1 Universidad de Buenos Aires. Facultad de Farmacia y Bioquímica. Departamento de Bioquímica Clínica, Cátedra de Bioquímica Clínica I, Laboratorio de Endocrinología. Buenos Aires, Argentina.; 2 Universidad de Buenos Aires. Facultad de Farmacia y Bioquímica. Instituto de Fisiopatología y Bioquímica Clínica (INFIBIOC). Buenos Aires, Argentina.; 3 Universidad de Buenos Aires. Facultad de Farmacia y Bioquímica. Departamento de Matemática. Buenos Aires, Argentina; 4 Universidad de Buenos Aires. Hospital de Clínicas “José de San Martín”. División Ginecología. Buenos Aires, Argentina.; 5 Universidad de Buenos Aires. Facultad de Farmacia y Bioquímica. Departamento de Bioquímica Clínica, Cátedra de Bioquímica Clínica II, Laboratorio de Fertilidad Masculina. Buenos Aires, Argentina.

**Keywords:** thyroid hormones, follicular fluid, oocyte, assisted fertilization procedures, follicular development, oocyte maturation

## Abstract

**Objective:**

Our aim was to analyze the role of thyroid hormones in follicular fluid (FF) in relation to the number of oocytes retrieved in women recruited for an assisted fertilization procedure.

**Methods:**

Retrospective cohort study of 51 women 37.5±3.3 years, range 29-42, evaluated after a controlled ovarian stimulation protocol in a University Hospital. FF was sampled by transvaginal ultrasound-guided aspiration after ovarian hyperstimulation and we measured T3 (T3f), T4 (T4f), TSH (TSHf) and free T4 (T4ff). The oocyte maturation rate was calculated as: Number of metaphase II oocytes/Number of oocytes retrieved x 100. Statistical analysis was performed using the SPSS-19 software.

**Results:**

Hormone levels in FF were: TSHf 1.3µIU/ml (0.4 - 2.7), T3f: 1.52±0.46 nmol/L, T4f 88.8±30.9nmol/L and T4ff: 15.44±2.57pmol/L. The number of oocytes recovered was dependent onT4f following the equation: Log (oocyte) = 0.379+0.042*T4f (r:0.352, *p*=0.012). After a logistic regression model analysis, T3f showed a tendency to be associated with the OMR: OR (95 % CI)= 0.977 (0.954 to 1.001), *p*=0.057.

**Conclusions:**

The correlation found between thyroid hormones and the number of oocytes retrieved suggests an interaction between thyroid and gonadal axes in relation to follicular development.

## INTRODUCTION

Assisted reproductive technology (ART) has grown by leaps and bounds in recent decades; the optimization of these procedures comes from basic reproduction physiology knowledge (Ishihara *et al*., 2015). In this sense the gonadal axis is explored to adjust the optimal stimulation schemes in assisted reproduction programs. However, the role of the thyroid axis in this process has been less studied, despite large evidence that emphasizes its importance in natural fertility (Vissenberg *et al*., 2015; Alemu *et al*., 2016).

In brief, thyroid hormones play an important role in conception and pregnancy, and are essential for normal adult health, fetus and childhood development (Alexander *et al*., 2017; Yassaee *et al*., 2014). Alterations in thyroid physiology can lead to menstrual irregularities, ovulation disturbances and therefore, reduced possibilities of a successful pregnancy (Vissenberg *et al*., 2015). However, their physiological mechanism in fertilization is not elucidated yet.

Many studies have shown an association between maternal hypothyroidism with obstetric complications and/or psychomotor impairment in the offspring (Committee of the American Society for Reproductive Medicine, 2015; Maraka *et al*., 2017). Although there is only limited evidence on the possible positive effects of T4 treatment in such cases, there is widespread agreement among clinicians about the need for treatment of clinical hypothyroidism during pregnancy (Velasco & Taylor, 2018). Oocyte maturation and embryo development are controlled by hormones as well as intra-ovarian factors such as cytokines and growth factors. In assisted reproduction the number of mature oocytes retrieved is a key point (Milachich & Shterev, 2016), although oocyte quality is more important than its quantity (Verberg *et al*., 2009).

It is well known that spermatozoa exposure to FF favors the acrosomal reaction, its mobility and ability to penetrate the ovum (De Jonge, 2017). In this sense, we have previously reported that in vitro addition of T4 stimulates sperm hyperactive movement, increasing the recovery rate after enrichment techniques like “swim up” (Mendeluk & Rosales, 2016). We were interested in evaluating the role of thyroid hormones in the female reproductive tract.

Several isoforms of thyroid hormone receptors mRNA were described in 1997 to be expressed in human oocytes, suggesting a probable hormonal direct effect either on the oocyte *per se* as well as on the granulose cells (Zhang *et al*., 1997). In an indirect way, the effect on cumulus cells may affect the oocytes as well. Recently, the enzyme involved in thyroid hormones biosynthesis, thyroid peroxidase, was revealed for the first time in granulose cells, supporting the hypothesis that the ovarian follicle is an independent thyroid hormone producing unit (Monteleone *et al*., 2017). Although iodine concentration in thyroid gland is higher than in other organs, ovarian uptake and buildup was also described. The physiological importance of this process is not yet completely known.

The process of oocyte maturation, a key point in fertilization, is highly related to its environment, the FF (Chang *et al*., 2016). By studying its composition, important information may be obtained to clarify the whole process. Our aim was to analyze the role of thyroid hormones in FF in relation to the number of oocytes retrieved in women recruited for an assisted fertilization procedure.

## MATERIALS AND METHODS

In a retrospective cohort study, we included 51 women of 37.5±3.3 years of age, ranging between 29-42; 11 women ≤35 years, 24 between 35 and 39 years and 16≥40 years. All of them were evaluated after a controlled ovarian stimulation protocol at the Gynecology Division, Hospital de Clínicas "José de San Martín". Universidad de Buenos Aires, Argentina. The inclusion criteria were: woman with infertility for more than 12 months before being included in the study, having regular menstrual cycles of 24-35 days, presumably ovulating, ultrasound visualization of both ovaries without evidence of abnormalities in their first treatment cycle, with FSH serum levels in the early follicular phase lower than 12 IU/l, antral follicle count (diameter 2-10 mm) greater than 2 for both ovaries, with no endometriosis nor diseases of genetic origin, and body mass index between 18-25kg/m^2^. To be included in the study, the women should have serum TSH levels and antithyroperoxidase antibodies within the reference range. Women with endocrine diseases, autoimmunity or medication affecting thyroid function were excluded. The study was approved by the Institutional Review Board of the Hospital. Informed consent was obtained from all individual participants included in the study.

Ovarian reserve was evaluated in all patients through anti-Müllerian hormone (AMH) levels and antral follicle count by ultrasound. Only one cycle per woman was selected for this study. The women were scheduled for controlled ovarian stimulation with three different protocols: recombinant human FSH (rhFSH) only (150-300 UI); rhFSH along with recombinant LH (75 UI) and rhFSH plus human menopausal gonadotrophin (HMG, 75-150UI). In all cases, the hormones were administered subcutaneously, daily from day 2 of the cycle. Treatments lasted between 7 and 10 days. In order to avoid endogenous LH peaks, the women received a Gn-RH antagonist (0.25 mg) from day 7 (± 1) of the menstrual cycle until ovulation induction. Human chorionic gonadotrophin (hCG 10000 UI) was administered subcutaneously to induce ovulation. Oocyte retrieval was performed 36 hours after hCG administration. ICSI was performed in all cases.

FF was sampled and a transvaginal ultrasound-guided aspiration of the hyperstimulated ovary was performed; each follicle was individually aspirated and collected. For each follicle, the presence or absence of an egg was recorded immediately under a stereoscope and the residual follicular fluid was placed into a 15 ml sterile Falcon conical tube. The FF was cleared by centrifugation at room temperature for 10 minutes at 300× g, aliquoted and placed at -80ºC for later analysis.

The remnant FF collected was thawed and T3 (T3f), T4 (T4f), TSH (TSHf) and free T4 (T4ff) were measured using chemiluminescence immunoassay on Advia Centaur XP autoanalizer. All oocytes retrieved were evaluated to analyze the complex cumulus corona expansion degree and the oocytes maturational stage was determined after denudation of oocytes by enzymatic and mechanical methods. Only those with a visible polar body were classified as mature or in metaphase II (MII). The oocyte maturation rate was calculated as: Number of metaphase II oocytes/Number of oocytes retrieved x 100. We employed a logistic regression model to determine whether a relationship exists between OMR and independent variables: T3f, T4f, TSHf, and T4ff. The response variable was coded considering an OMR cut-off value ≥ 60.

### Statistical Analysis

We ran the statistical analysis using the SPSS-19 software, considering values of *p*<0.05 statistically significant. The results are expressed as mean±SD or median (range) according to data distribution. The differences among treatment groups were assessed by Kruskal-Wallis ANOVA. The number of oocytes retrieved in relation to the different variables (T3f, T4f, TSHf, and T4ff) was evaluated by multiple regression analysis. We used logistic regression to determine if any of the hormones tested was associated with OMR.

## RESULTS

Table 1 shows women`s mean ages, treatment used for each group of patients, the number of oocytes retrieved, the number of metaphase II oocytes and infertility etiology.

**Table 1:** Characteristics of the studied population according to treatment and results obtained

Treatment	*rhFSH* n=11	LH-*rhFSH* n=30	HMG-*rhFSH* n=10
Mean age (years)	34.7±3.1 (29-39)	38.1±3.2 (29-42)	38.7±2.0 (34-41)
Male factor	5	3	2
Tubal factor	1	10	1
Decreased ovarian reserve	1	0	0
Mixed	4	15	6
Idiopathic	0	2	1
No of Oocytes retrieved	6.9±3.8 (2-13)	5.1±3.7 (0-18)	5.4±2.9 (3-12)
No of Oocytes MII	4.7±2.2 (2-8)	3.2±2.4 (0-12)	2.9±2.2 (0-7)

*rhFSH*: recombinant human FSH;

*rhFSH*: rhFSH along with recombinant LH;

*rhFSH*: rhFSH plus human menopausal gonadotrophin: MII: metaphase II oocytes

The number of oocytes retrieved and the number of oocytes in MII were not significantly different among the three treatments groups, neither among the three age groups: ≤35, 35 -39 and ≥40 years (Kruskal-Wallis ANOVA).

Serum TSH levels were 1.8µIU/ml (0.4-4.0). Hormone levels in FF were: TSHf: 1.3µIU/ml (0.4 - 2.7), T3f: 1.52±0.46nmol/L, T4f: 88.8±30.9nmol/L and T4ff: 15.44±2.57pmol/L.

The number of oocytes recovered was 5 (0-18), and the number of oocytes in metaphase II was 3 (0-12), in both cases the data are expressed as median (range).

There was only one cancellation in which case no oocytes were recovered. In order to determine if there is any relation among the number of oocytes retrieved and the following independent variables: T3f, T4f, TSHf, T4ff, we performed a multiple regression analysis. We found that the number of oocytes recovered was only dependent on T4f. As the assumption of normality was not met, a logarithmic transformation of the dependent variable (log (oocyte) was carried out. The resultant equation was as follows:

Log (oocyte) = 0.379+0.042 * T4f (r: 0.352, *p*=0.012)

The median of OMR was 66, ranging from 57 to 74. In order to evaluate if thyroid hormones were related with OMR, we applied a Logistic Regression Model. T3f showed a tendency to be related with the OMR: OR (95% CI) = 0.977 (0.954 to 1.001), *p*=0.057. No relationship between OMR and T4f, TSHf and T4ff was found.

## DISCUSSION

The thyroid axis is currently evaluated in women entering a fertilization program, since an euthyroid state is mandatory to reach successful outcomes. In this way, hypothyroid women are supplemented with levothyroxine in order to reach an euthyroid status. These arguments explain the well-known idea that the hypothalamus-hypophysis-gonadal axis plays a major role in fertilization, while the thyroid axis has a facilitating one (Colicchia *et al*., 2014). Less is known about the molecular mechanisms involved in thyroid hormone actions in this process.

Thyroid hormones seemingly influence the maturation of human oocytes (Vissenberg *et al*., 2015), their receptors have been isolated in mural granulose and cumulus cells and the mature oocyte of the human ovarian follicle (Xie *et al*., 2010). Enzymes involved in the chain that regulate the generation of thyroid hormones have also been found in granulose cells (Monteleone *et al*., 2017). Many reports show the presence of thyroid hormones and their receptors in FF, stating that they would be involved in human endometrial physiology through a probable positive role during folliculogenesis and ovulation (Colicchia *et al*., 2014).

Knowledge about the influence of thyroid hormones on reproduction is being applied to animal production. In this sense, Costa *et al*. (2013) hypothesizes that T3 may have a beneficial effect on the kinetics of embryo development in bovines. In our study we demonstrated that T3f should be a predictor of OMR≥60. This OMR cut-off value is which in our experience has clinical value, although other authors use slightly higher values (Abbara *et al*., 2018).

Recent studies have revealed that thyroid hormones alter estrous cyclicity and antioxidative status in the ovary of the rat acting through the nitric oxide synthase signaling pathway (Zheng *et al.,* 2015; Wei *et al*., 2018). It was also reported that ovarian follicles of the laying hen express mRNAs of thyroid hormone-nuclear receptors, as well as integrin (αVβ3) plasma membrane receptors, indicating a genomic and non-genomic action of thyroid hormones in the chicken ovary (Sechman, 2013).

Data reported in the literature support the idea that thyroid hormones would play a direct role in ovulation, early follicular development, differentiation and stimulation of steroidogenic capacity of granulose cells (Vissenberg *et al*., 2015). Thyroid hormones are considered biological amplifiers of the gonadotropins stimulatory action. In combination with FSH, thyroid hormones increase the proliferation and inhibit the apoptosis of these cells by the PI3K/Akt pathway (Vissenberg *et al*., 2015; Monteleone *et al*., 2017). Thyroid hormones may play a key role in the regulation of reproductive processes (Cedíková *et al*., 2012). Our study agrees with the above statement through the analysis of human FF in order to contribute to the knowledge about human ovarian function and disorders related to the reproductive process.

Different results have been reported while comparing thyroid hormone levels in serum and FF in humans and animals. Wakim *et al*. (1993) found that T3 and T4 levels in FF were similar to serum values, with a positive correlation between serum and FF T4 values in humans. In turn, Slebodziński (2005) refers lower values for T4 and within the normal range or higher for T3 in serum *vs.* FF in animals. FF values in our patients are similar to those in serum concentrations. Although we could not measure thyroid hormones in the serum, our results are in agreement with those reported by Wakim *et al*. (1993) and Cedíková *et al*. (2012).

According to the standards of our Hospital Ethics Committee, we can only obtain FF from infertile women who enter an ART program, so we did not manage to get a group of fertile women to compare with the infertile ones. Nevertheless, we consider that this is not mandatory, taking into account that the aim of the study was to report our findings concerning thyroid hormones in FF in association with the number of oocytes retrieved in assisted fertilization procedures.

One limitation of this study is the lack of serum T4 levels. However, due to clinical data and considering that TSH levels and anti thyroperoxidase antibodies were within the reference range, we assume euthyroid condition in all women studied. We must also take into account the wide range of patient’s ages that could be the cause of the large variation observed in the mean number of recovered and mature oocytes.

To our knowledge, our study is the first to report a correlation between T4 in follicular fluid and the number of oocytes retrieved in an assisted reproductive program, based on a mathematical equation determined in our population, which reflects a biological event. This evidence suggests an interaction between thyroid and gonadal axes, in relation to follicular development and oocyte maturation. Given that the critical events of oocyte and follicular maturation take place in a follicular fluid environment, a thorough identification of the specific components that are involved in this process is mandatory. Prospective studies with larger number of patients should be carried out to check our results.
